# The Easy and Versatile Neural Recording Platform (T-REX): Design and Development Study

**DOI:** 10.2196/47881

**Published:** 2023-10-24

**Authors:** Joaquín Amigó-Vega, Maarten C Ottenhoff, Maxime Verwoert, Pieter Kubben, Christian Herff

**Affiliations:** 1 Computer Science Department Gran Sasso Science Institute L'Aquila Italy; 2 Neurosurgery School for Mental Health and Neuroscience Maastricht University Maastricht Netherlands

**Keywords:** recording, platform, flexible, data recording, neurotechnology, experiments

## Abstract

**Background:**

Recording time in invasive neuroscientific research is limited and must be used as efficiently as possible. Time is often lost due to a long setup time and errors by the researcher, driven by the number of manually performed steps. Currently, recording solutions that automate experimental overhead are either custom-made by researchers or provided as a submodule in comprehensive neuroscientific toolboxes, and there are no platforms focused explicitly on recording.

**Objective:**

Minimizing the number of manual actions may reduce error rates and experimental overhead. However, automation should avoid reducing the flexibility of the system. Therefore, we developed a software package named T-REX (Standalone Recorder of Experiments) that specifically simplifies the recording of experiments while focusing on retaining flexibility.

**Methods:**

The proposed solution is a standalone webpage that the researcher can provide without an active internet connection. It is built using Bootstrap5 for the frontend and the Python package Flask for the backend. Only Python 3.7+ and a few dependencies are required to start the different experiments. Data synchronization is implemented using Lab Streaming Layer, an open-source networked synchronization ecosystem, enabling all major programming languages and toolboxes to be used for developing and executing the experiments. Additionally, T-REX runs on Windows, Linux, and macOS.

**Results:**

The system reduces experimental overhead during recordings to a minimum. Multiple experiments are centralized in a simple local web interface that reduces an experiment’s setup, start, and stop to a single button press. In principle, any type of experiment, regardless of the scientific field (eg, behavioral or cognitive sciences, and electrophysiology), can be executed with the platform. T-REX includes an easy-to-use interface that can be adjusted to specific recording modalities, amplifiers, and participants. Because of the automated setup, easy recording, and easy-to-use interface, participants may even start and stop experiments by themselves, thus potentially providing data without the researcher’s presence.

**Conclusions:**

We developed a new recording platform that is operating system independent, user friendly, and robust. We provide researchers with a solution that can greatly increase the time spent on recording instead of setting up (with its possible errors).

## NeuroTech Dialogue

We propose a software package called T-REX (Standalone Recorder of Experiments) that is specifically designed for recording experiments. T-REX automates multiple manual actions, reducing the experimental overhead and error rate during recordings. With our system, researchers can centralize all their experiments into a simple local web interface, and set up, start, and stop experiments with a single button press. The user friendly interface can be used with different recording modalities, amplifiers, and participants, making it highly flexible. The software is executable on mainstream operating systems (Windows, Linux, and macOS) and does not require the use of a specific programming language for creating the experiments. It includes functionality to automatically record experimental data using a protocol frequently used in the community called Lab Streaming Layer. With T-REX, we simplify and streamline the recording of experiments for researchers while providing maximum flexibility in using different recording modalities, programming languages, operating systems, and amplifiers.

## Introduction

Recording high-quality electrophysiological human brain activity is notoriously difficult. The best quality signal has both high spatial and temporal resolution and is recorded with invasive electrodes [1,2]. However, since implanting electrodes in humans for research purposes is a lengthy and challenging process with many safety and ethical concerns, scientists tend to use the clinical treatment of patients who receive implants for clinical purposes [3,4] as a research vehicle. Some examples are patients with medication-resistant epilepsy undergoing presurgical monitoring for resection surgery [5] or patients qualified for deep brain stimulation [6].

Because recordings should not interfere with clinical treatment, the time to record data for neuroscientific experiments in these patient groups is severely limited. For implanted epilepsy patients, the recording windows are usually a few days to 2 weeks. In contrast, for patients with deep brain stimulation, the recording windows are during surgery using microelectrode recordings, and between surgery and when the stimulator is turned on. During these recording windows, patients need time to recover and have sufficient general well-being to participate. Moreover, time spent on clinical treatment and other assessments that require recording time can further reduce the already limited recording time.

Therefore, the brief remaining time window should be used as efficiently as possible. In practice, this means that the time spent on recording should be maximized, while the time spent on setting up and solving errors should be minimized. Both the set-up time and error rate can be significantly reduced by automating as many manual actions as possible (eg, connecting to recording devices; starting experiments; selecting data streams; and starting, stopping, and synchronizing the recording). However, as experiments or recording setups change over time, it is often not worthwhile for research groups to invest in developing a more sophisticated system. It takes human resources, technical knowledge, and substantial time investment to move beyond custom-made systems, which are often only used internally and unavailable to the public. Aside from custom-made setups, there exist multiple measurement platforms, including BCI2000 [7], OpenVIBE [8], FieldTrip [9], NFBlab [10], and MEDUSA [11]. These systems can record data from many different amplifiers and include modules to design, analyze, and provide feedback during or after the experiments. While all these platforms also include good recording capabilities, they are more broadly focused on experimental design and analysis.

Additionally, these solutions limit the experiments that can be executed by the researcher in some way, either by targeting a specific type of experimental design or by imposing some hardware or software tool sets, such as programming language, input/output devices, or operating systems (OSs). Furthermore, not all platforms are open-source, which is not in the spirit of open science and impedes collective quality control and replicability. For example, FieldTrip requires the researcher to use the proprietary platform MATLAB, and BCI2000 and OpenVIBE impose the use of their tools and application programming interfaces. Additionally, the researcher must install a complete software package on the system, even when only the recording functionality is needed. Ashmaig et al [12] developed and described a system exclusively focused on continuous data recording for neurosurgical patients. The system provides a good use case for naturalistic long-term recordings but has an extensive list of hardware requirements and limits the researcher to Linux. Furthermore, not all research groups have the opportunity to perform long-term recordings.

While all these platforms provide good solutions for their use case and cover a significant part of the neural recording space, we observed that none of these platforms are specifically tailored to the setup and recording of experiments. Here, we describe the T-REX (Standalone Recorder of Experiments) platform that is specifically targeted to improve the recording of experiments. By automating the setup, start, and stop of experimental recordings, T-REX reduces the error rate and time spent between recordings. T-REX minimizes restrictions on hardware and software, is available on all major OSs, and is publicly available as an open-source project. This work presents T-REX’s system design, functionality, usage, and potential implications for the field.

## Methods

### Requirements

We determined 3 criteria that the system should meet to make T-REX applicable to as many labs as possible. First, T-REX should be as independent as possible of tools, paradigms, OSs, and programming languages. Each lab has its preferred tool set, and ensuring independence means that researchers do not need to port their existing experiments to fit T-REX. Its only requirement is for the experiments to use Lab Streaming Layer (LSL) to stream data [13]. The backend of T-REX uses LSL to synchronize data across sources (see the section Details of LSL). Second, T-REX should be user friendly to both the researcher and the participant. Increasing simplicity will reduce error rates and the time spent on setting up, which can be achieved by automating multiple manual actions. Lastly, the system should be robust. This means that an experiment should only run when all requirements to run are met, and in case of technical problems, the experiment should retain the data up to that point and return to the *Home* screen.

### System Outline

**Figure 1:**
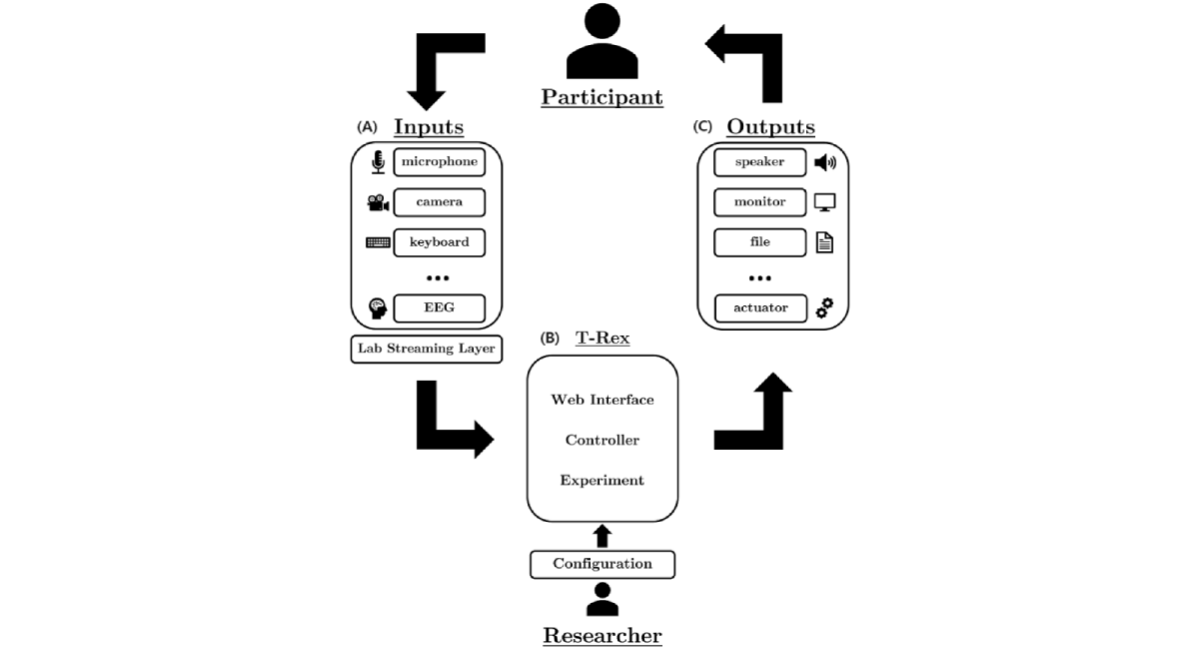
A schematic overview of the experiment loop of T-REX (Standalone Recorder of Experiments). (A) Data from the participants (eg, EEG, movement, and audio) are recorded by a variety of device inputs. Each input device should create a Lab Streaming Layer StreamOutlet to make the data available to record. (B) T-REX then provides a user interface for experiment selection. The backend finds the required data streams and records them. The rounded box shows the different software components (web interface, controller, and user configuration). (C) Example outputs of the experiment. These components interact with the participant (experiment user interface and stimuli), or the recorded data are saved. EEG: electroencephalography.

**Figure 2:**
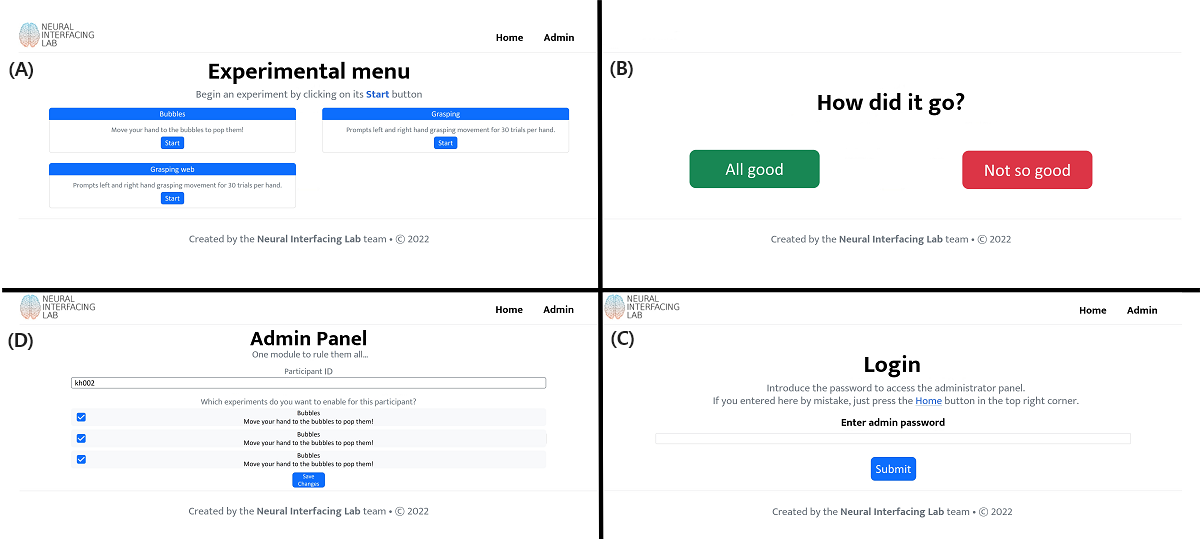
Representation of the main 4 windows of the web interface. (A) The Home window contains all the experiments accessible to the researcher, represented on a grid configuration. (B) The Experiment Feedback window allows obtaining feedback from the participants about their experience with the experiment. It is achieved through the green (“All good”) and red (“Not so good”) buttons. Participants can only continue after pressing one of these buttons. (C) The Admin Login window allows access to the administration panel by entering the password. (D) The Admin Configuration window allows the administrator to create new participants and modify their access to experiments.

In brief, T-REX acts as the middleman handling the experimental overhead for the researcher (Figure 1). When using T-REX, the researcher can select an experiment by pressing a button on the main menu screen (Figure 2). T-REX will then check the availability of all required data streams and connect to the streams. Examples of data streams include a hand-tracking device sending coordinates of a person’s hands and an amplifier recording the participant’s neural activity. T-REX will then start the experiment user interface (UI) that instructs the participant on what task to perform. Upon successful start of the experiment UI, T-REX starts recording all data streams and saves them to a folder specified by the researcher. All data are saved by LSL into a single .xdf file. After the experiment is completed, the UI prompts the participant on how the experiment went and returns to the *Home* screen. During the full experiment loop, the actions that the researcher needs to perform are to start the required device data streams and select the experiment in the *Home* screen.

### Materials, Software, and Technologies

T-REX has multiple components, including a local web interface, a recording backend, and a controller interface connecting these 2 components. The web interface (Figure 2A-D) is built using Bootstrap5 [14] for the frontend and the Python package Flask [15] for the backend. The recording backend uses LSL and handles data stream synchronization and recording itself (information is provided in the section Details of LSL). Lastly, the controller interface (information is provided in the section Controller) is implemented in Python 3.7+ and a few dependencies found in requirements.txt. T-REX is compatible with Windows, Linux, and macOS.

### Details of LSL

T-REX uses LSL to synchronize the data streams from different devices, such as a variety of electroencephalography (EEG) amplifiers, audio streams, movement trackers, and cameras. The service handles “networking, time-synchronization, (near) real-time access, and optionally the centralized collection and recording of data” [13]. It is lightweight and has multilanguage and multiplatform support, including Unity and Android. LSL allows the researcher to send data via a data stream to a local network server, which can be recorded.

Basic usage involves defining a StreamOutlet that makes a time series data stream available on the network. The data are pushed per sample or per chunk into the outlet. By creating an outlet, the stream is made available to the local network of computers. The most basic usage (in Python) is represented in the following code block:



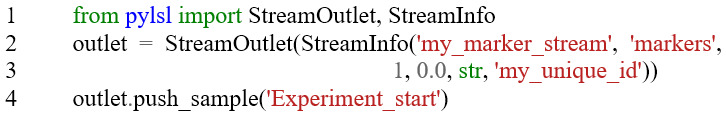



This code creates a StreamOutlet object with a name (“my_marker_stream”), type (“markers”), channel count (1), irregular sample rate (defined as 0.0), data type (“str”), and source ID (“my_unique_id”). Lastly, a sample containing “Experiment_start” is pushed to the outlet.

Inversely, to receive data, one can instantiate a StreamInlet and use inlet.pull_sample(). For a comprehensive overview, see the official documentation [13]. For T-REX to be able to record all data, the devices and the experiments themselves must all create a StreamOutlet (like the example above). If no StreamOutlet is created, T-REX will not be able to find and record the device and start the experiment. By using LSL, T-REX is able to connect to many popular experiment platforms, such as Psychopy [16], OpenSesame [17], and Presentation [18]. In case a stream is listed in the requirements provided by the config in an experiment but is not available, T-REX will throw an error and return to the *Home* screen. Thus, no experiment can start while missing a data stream.

### Trigger

In some recording setups, a trigger marks the start and end of an experiment. In these setups, participants’ clinical data are recorded continuously and stored on a server. During an experiment, the data cannot be streamed directly and need to be retrieved afterward by the responsible data steward. The data steward can locate the requested data files by identifying the trigger pattern sent by the experimenter. Depending on the manufacturer, a trigger can be delivered via the amplifier or with a separate device. If it can be delivered internally, the experimenter can directly send triggers from within the experiment, and the trigger functionality of T-REX does not need to be used. T-REX provides some basic functionality to send a trigger code if an external device is required. In short, T-REX searches for a USB device with a name set in the main configuration file. It connects to this device and sets up an LSL stream. Then, if an experiment is started and the trigger flag in the main configuration file is set to True, the trigger class sends a user-defined code. When the experiment is finished, the trigger will be sent again, flagging the start and end of the complete experiment. The data steward can then retrieve the correct data with these trigger codes. At the same time as sending a trigger, the code also sends a marker to LSL, allowing for synchronization across data streams.

### Software Components

The software consists of 2 main components: the *web interface* that handles the UI and the *controller* that sets up, starts, and stops all experiments (Figure 1B).

### Web Interface

The web interface includes 4 windows: *Home*, *Experiment Feedback*, *Admin Login*, and *Admin Configuration* (Figure 2).

The *Home* window (Figure 2A) displays all the experiments in a grid. Experiment cards are shown on that grid with a title, description, and start button. When the button is pressed, the controller executes a command that starts the selected experiment. The command is defined by the researcher and specified on the configuration of the experiment (more details are provided in the section User Configuration). During the experiment, the web interface is on standby awaiting the completion of the experiment.

After completion, the participant is redirected to the *Experiment Feedback* window, where the question “How did the experiment go?” is prompted (Figure 2). The participant or researcher is required to select a feedback option to continue. This allows the researcher to save a brief experiment evaluation to assess data quality in later analysis. In potential future applications, the participants might perform the experiments by themselves. Then, this feedback is useful to flag the researcher to be aware of potential poor data quality. The feedback is stored under the file name feedback.txt in the same folder as the most recent .xdf file (that contains the data recorded from the experiment).

The *Admin Configuration* provides the researcher with a closed environment where the participant identifier can be selected and a selection of all available experiments is available. To access the *Admin Configuration*, the researcher must first log in using the password that is configured in the main configuration file (Figure 2C; details are provided in the section User Configuration). When logged in, the researcher can see the configuration of the active experimental session, composed of an alphanumeric participant identifier and their access to experiments. A list of all the experiments included in the platform is visible from this window, but only those with checked marks are visible to the participant. The changes in this window are only applied after pressing the “Save” button at the end of the page.

The web UI has been tested with Firefox (version 105.0.1), Chrome (version 106), Safari (version 16), and Edge (version 106), although it should be compatible with higher versions and other mainstream browsers.

### Controller

**Figure 3:**
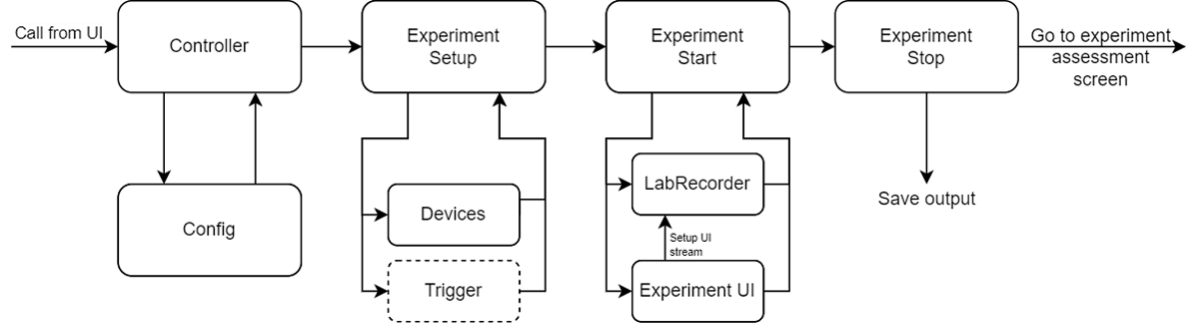
Backend flow of running an experiment. When an experiment is started by pressing the start button on the card, the controller is called, loading the main configuration file and extracting the information received from the user interface (UI) about which experiment to run. Then, an experiment instance is created, loading the experiment-specific information and completing the setup in 3 steps. First, it checks for all devices and their Lab Streaming Layer streams. Second, it initializes a recorder instance and adds all streams to the list of streams it should record. Lastly, if a trigger is required for the selected experiment, it will set up a trigger class that searches and connects to the trigger. Once the subprocess call is returned, experiment sends the final trigger and stops the recorder. The data are saved in the ./output/ folder, and the researcher or participant is redirected to the experiment assessment screen (Figure 2B).

The controller handles everything related to running an experiment and has 3 main parts: setup, start, and stop (Figure 3). The related code can be found in the ./libs directory.

#### Setup

When an experiment is started by pressing the start button on the card, the controller class in *Controller.py* (Figure 3) is called, and it loads the main configuration file and extracts the information received from the UI about which experiment to run. With this information, an *experiment* instance is created, and its loading function is called.

*Experiment* loads the experiment-specific information and completes the setup in 3 steps. First, it checks for all devices and their LSL streams as defined by the researcher in the experiment configuration under device_inputs.

Subsequently, *experiment* initializes a *recorder* instance and adds all streams to the list of streams it should record. For a movement experiment [19-23], the streams recorded could be the neural amplifier and experimental triggers. Additionally, a movement tracker [24-26] or a force sensor [27] could be added. For speech perception [28-30] or auditory perception [31,32], the audio stream, experiment triggers, and neural data need to be recorded. For speech production [33-36], the streams could be neural data, microphone, and triggers. In the Results section, we provide some example experiments.

The last step is to check if a trigger is required for the selected experiment. If so, it will set up a trigger class that searches and connects to the trigger.

All devices must be connected and available to LSL before the *experiment* instance is called. As all requested devices are essential for successful recording, T-REX will raise an error and return to the UI if not all input devices are connected successfully.

#### Start

A user-defined command is called using Python’s subprocess library to start the experiment UI. The command should be callable from the command line interface and can be set in the experiment-specific configuration. Because the experiment UI likely contains a stream that sends out experiment-related markers, *experiment* will start a loop on a user-defined timeout to search for the marker stream. Once found, usually almost instantly, the *recorder* will start recording all streams. Implementing the system this way does not restrict the research aside from using LSL. However, owing to the timeout, the experiment may start before the recording starts. This can only happen if the time between the setup of the experiment StreamOutlet and sending the first marker is shorter than the time that the *recorder* can find the stream and start the recording. Usually, finding the StreamOutlet and starting the recording is in the order of milliseconds. However, to entirely prevent the possibility of this happening, we recommend including a waiting screen in the experiment UI (eg, “Press button to start”) or ensuring sufficient time (longer than the timeout set in the experiment configuration) between the setup of a StreamOutlet and the start of the experiment. Once connected to the experiment StreamOutlet, the experiment UI should start, and the *experiment* instance will wait until the called command is terminated and returned, which usually happens when the experiment UI window is closed.

#### Stop

Once the subprocess call is returned, *experiment* sends the final trigger and stops the *recorder*. The data are saved in the ./output/ folder, defined in the main configuration file (information is provided in the section User Configuration). An example of the created directory tree is provided in Multimedia Appendix 1.

### Device Inputs

Each experiment can have multiple input devices, such as an amplifier measuring the neural data, a hand-tracking device, and a microphone. Any device can be included if it generates a StreamOutlet. Each device should send the data from the device to LSL, allowing it to be accessed by the other system components and to be recorded. The name, type, or source_id supplied to the StreamOutlet will be the values that T-REX will search for during experiment setup (information is provided in the section Controller). In practice, this means that either the name, type, or source_id needs to be supplied under device_inputs in the experiment configuration file (information is provided in the section Experiment Configuration). Since devices can be used for multiple experiments, we included a separate destination for all device input files (./exp_module/inputs), although input devices can be stored anywhere as long as they generate a StreamOutlet.

### User Configuration

There are 2 types of configuration files that the researcher can set: main configuration and experiment-specific configuration. All configuration files are formatted in Yet Another Markup Language (YAML).

#### Main Configuration

The file config.yaml in the root folder contains the system-wide configuration. This configuration file contains information on general settings. Multimedia Appendix 2 provides a description of the different available options, and Multimedia Appendix 3 provides an example of the main configuration file. The main option under *path* is the path that all relative paths will be anchored to and should be set to the root folder. Most parameters are preset, but out and trigger configurations may vary between different recording setups and might need to be redefined.

#### Experiment Configuration

Each experiment included in T-REX requires a separate folder in ./exp_module/experiments/ and must include at least 2 files: config.yaml and the file to start the experiment. A full description of all the fields and different options in config.yaml can be found in Multimedia Appendix 4. The *name* and *description* define the text shown in the UI; *command* sets the command line interface command made by the controller class to start the experiment; and *exp_outlet* sets the name, type, or source_id that the experiment class will search for. For example, if the experiment UI is a Python script that will create a StreamOutlet named markers, the *command* to execute would be python .\exp_module\experiments\your_experiment_file.py and *exp_outlet*=’markers’.

## Results

### Overview

We have included 3 different example experiments to provide a practical view of how to use T-REX. The examples can also serve as a quick start for researchers to create new experiments or adapt the ones included. A step-by-step explanation of adding a new experiment is described in the section Adding New Experiments to the Platform.

### Case 1: Simple Experiment in Python

**Figure 4:**
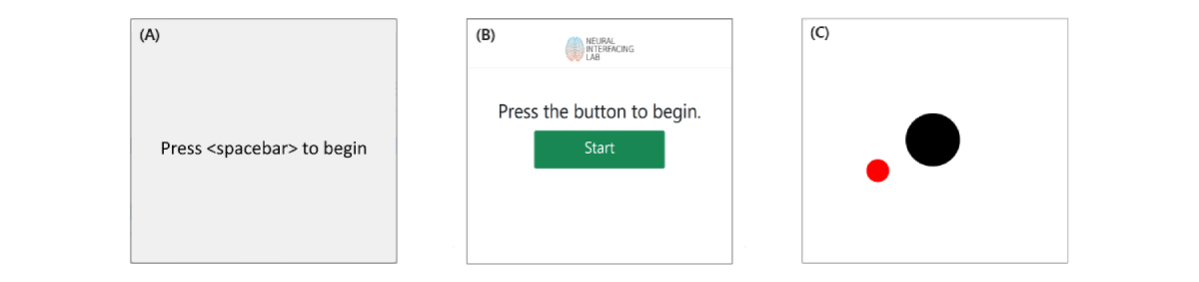
User interfaces for the 3 use case experiments included. (A) Grasping: simple text-based experiment built using the Python package Tkinter. (B) Grasping web experiment: reimplementation of the grasping experiment as a single page application (SPA) to allow its execution on any device with access to a web browser. (C) 3D hand-tracking experiment: the hand-tracking is performed using the LeapMotion controller, and the experiment is implemented in Python using the package Tkinter.

This experiment is a simple text-based instruction for a grasping task (Figure 4A). The participant is prompted by text in a Python Tkinter [37] window to continuously open and close either the left or right hand, as used previously [38]. The experiment requires neural data as the input device and generates a StreamOutlet to send markers that inform about the start and end of the experiment and of the trials. The neural data are acquired from a stream with *name*=*Micromed*, *type*=*EEG*, and *source*_*id*=*micm*01. These values are all set by the researcher. As T-REX will search for all 3 options (name, type, and source_id), only 1 must be provided. Therefore, the option under device\_inputs in grasping\config.yaml is set to eeg (case insensitive). Next, the *marker* StreamOutlet that will be generated by the experiment has *source*_*id*=*emuidw*22. When the *experiment* class runs the experiment command (command field in grasping\config.yaml), it will search for these streams. Therefore, the exp_outlet field is set to ’emuidw22’. Finally, since the grasping experiment is Python-based, the command should use Python to call the script with the command: python .\exp_module\experiments\grasping\grasping.py. The configuration file used has been presented in Multimedia Appendix 5.

When these options are set, the experiment is ready to go and can be started by pressing the start button on the *Home* window. The Tkinter window opens and waits for the spacebar to be pressed. Once pressed, the experiment starts and is locked as the top viewed window until completion. When the experiment is finished and closed (ie, the command call ends and returns to the *experiment* class), the *experiment* instance stops the recording and saves the data. In-depth details on how experiments are started and stopped are described in the section Controller.

**Figure 5:**
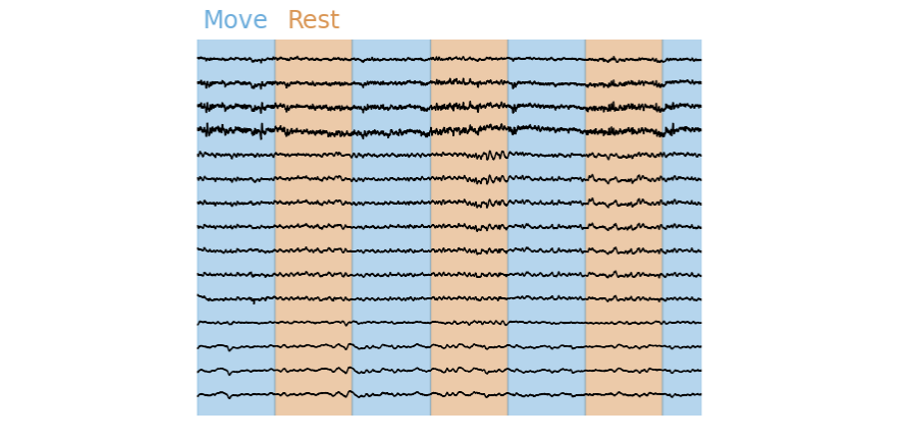
Neural data were recorded with the grasping experiment using T-REX (Standalone Recorder of Experiments). Two streams were recorded during this experiment: an EEG stream and a marker stream. The data from the EEG stream are shown by the black lines, indicating the voltage over time in a selection of 15 neural electrodes. The marker stream sends the start and end of the experiment and the individual trials. These markers were used to determine the colored areas (blue and orange) shown. EEG: electroencephalography.

Figure 5 shows a random selection of 15 channels of neural data recorded with T-REX during the grasping experiment. Two streams were used in this experiment. First a *marker* StreamOutlet that sends all experiment-related markers, such as the start and end of the experiments and the start and end of each trial, with the accompanying label (move or rest). Second, an EEG StreamOutlet that streams the data from our Micromed Amplifier to LSL. With T-REX, these streams were automatically identified and recorded. The start and end of the colored columns (identifying move and rest trials) were determined by the recorded markers sent through the marker StreamOutlet. The synchronization by LSL ensures that the EEG and *marker* stream timestamps are the same.

### Case 2: Simple Experiment in a Web UI

We included the same grasping experiment as in Case 1 but implemented it in a web interface (Figure 4B). It uses a single page application (SPA) locally and thus can be created on any device with access to a web browser, like a laptop, tablet, and smartphone. The grasping web experiment also illustrates options other than a Tkinter window for experimenting. No internet connection is required, relieving some security concerns that could render execution on the web unsafe.

We constructed the experiment using HTML, CSS (Bootstrap5 for responsiveness and other visual aspects), and JavaScript for behavior. The device input is the same as in the Tkinter implementation of the experiment and the StreamOutlet containing the markers; thus, the device_inputs and exp_outlet are the same. The difference is in the command executed to start the experiment. In this case, start .\exp_module\experiments\graspingWeb\index.html is used. The configuration file used has been presented in Multimedia Appendix 6.

Once the experiment is started on the *Home* window, the *experiment* instance opens another tab on the browser displaying the “grasping_web” experiment. The experiment starts when the participant presses the green “Start” button. When the experiment is finished, the participant or researcher is prompted to press a red button to close the experiment. The GraspingWeb command call is finished at the button press and returns to the *experiment* instance, stopping the recording and saving the data.

### Case 3: Multiple Devices

**Figure 6:**
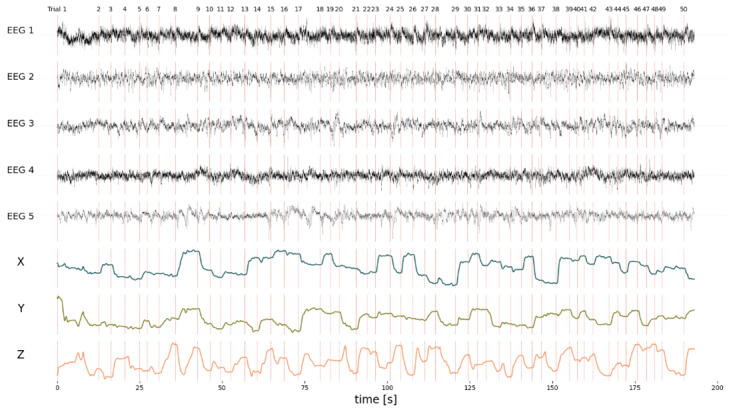
The combined data recorded from 3 different streams: an EEG stream, a marker stream, and a LeapMotion controller. The EEG channels are 5 channels randomly selected from 87 available channels. X, Y, and Z are the 3D coordinates of the palm of the hand, provided by a LeapMotion controller. The marker stream provides the shown trials (numbers on top with vertical dashed lines). To start and record this experiment, the LeapLSL stream has to be started, along with the EEG stream. Then, only the experiment needs to be started in T-REX (Standalone Recorder of Experiments). T-REX records all 3 streams (synchronized by Lab Streaming Layer), ultimately allowing to combine the 3 streams into this image. EEG: electroencephalography.

Lastly, we included a 3D hand-tracking experiment, where the goal is to hold a cursor (a black circle) on a target (a red circle). The cursor can be moved in 3 dimensions, where the third dimension controls the size of the circle (Figure 4C). In this case, the hand tracking is performed by the LeapMotion controller [39], but any other device can be used. We have provided a .exe file that reads the data from the tracker and sends it to an LSL StreamOutlet with *name*=*LeapLSL*, *type*=*Coordinates*, and *source*_*id*=*LEAPLSL*01. In addition to the hand-tracking information, we also need neural activity, for which we use the same StreamOutlet as described in Case 2. Lastly, the experiment is implemented in a Python Tkinter window and generates a marker stream similar to the stream described in the previous use case with *Source*_*id*=*BUBBLE*01. Thus, to set up the configuration for this experiment, we set the command to python .\exp_module\experiments\Bubbles\bubbles.py, exp_outlet to BUBBLE01, and device_inputs to LEAPLSL01 (the tracking information stream) and eeg (the neural data stream). To run the experiment, the researcher should start the device stream before the experiment is started in the *Home* screen (ie, run the .exe first). The configuration file used has been provided in Multimedia Appendix 7. An example of data recorded with T-REX for this experiment can be appreciated in Figure 6.

### Mix and Match

We have presented only 3 examples showing different possibilities. Different devices can be included by adding a StreamOutlet name, type, or source_id to the list of device_outputs. The only requirement to add a device is that the data from the device can be sent to a LabStreamingLayer StreamOutlet. This code is either supplied by the manufacturer or written by the researcher. If this requirement is met, any medical device or technology can be included, as T-REX does not impose any further restrictions on technologies or types of experiments, including, but not limited to, speech production, audio or speech perception, movement, decision-making, and simple or naturalistic tasks [40,41]. For example, new experiments can also be built in Unity [42] or PyGame [43] to provide better graphical experiences.

### Adding New Experiments to the Platform

The following steps describe how to add a new experiment from scratch to T-REX:

1. Create the experiment folder inside the directory ./exp_module/experiments/. An example of the directory tree for different example experiments can be found in Multimedia Appendix 8.
2. Create the experiment configuration file (config.yaml) inside the new folder. Information in Multimedia Appendix 9 can be used as the base example for creating this file, and the section Experiment Configuration contains a detailed description of each parameter.
3. Adjust the fields to the specific experiment.

After completing these initial steps, the experiment should be visible from the *Admin Configuration* panel. The researcher can set the experiment as “visible” from the admin panel by selecting its corresponding check mark. If configured as “visible,” it should appear on the *Home* window, and it can be executed by clicking on its respective button.

It is worth mentioning that when porting an already configured version of T-REX to a different OS, some parameters might need to be revised. For example, regarding the parameter command, when used on Windows to start a Python experiment, the definition is as follows:



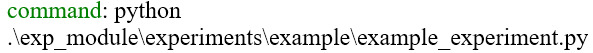



However, when used on Unix or Unix-like systems, the definition changes to the following:



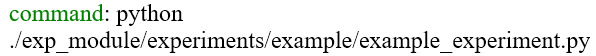



The difference comes because “/” is the path separator on Unix and Unix-like systems, and Microsoft uses “\”.

There might be other scenarios where the parameter command might differ between OSs; thus, we recommend revising each experiment configuration file when porting the platform to a different OS.

### Practical Experience

At the time of writing, we entirely switched to recording with T-REX for our experiments at different recording sites. So far, we have recorded multiple experiments, involving speech, motor, and decision-making tasks. Furthermore, at one of the recording sites, we recorded using the trigger functionality included in T-REX. We see no indications of different data quality in our neural decoding endeavors. We can decode speech [44,45] and movement trajectories [46] with performance equal to that using our previous setup.

## Discussion

We presented T-REX, an independent, user friendly, and robust system that minimizes the setup time and error rate. T-REX provides a simple UI and reduces the experimental setup to the press of a button. The software merges the LSL recording backend with a simple UI, automating experimental overhead for the researcher. T-REX reduces the setup time and error rate, resulting in more time spent recording neural data.

The simplicity of T-REX reduces the number of actions that the researcher must perform to only 2: starting the required devices and starting the experiment. The fewer manual actions the researcher needs to perform, the lower the chance that an error is made. It improves reliability and increases total data volume and time spent on recording. The LSL software package fully handles synchronization and recording. We decided on LSL as it is lightweight, is easy to use, has submillisecond timekeeping, and has a proven track record [47]. The flexibility of T-REX makes the system applicable in fields other than the neuroscientific context described here.

T-REX provides benefits for both the researcher and participant. A streamlined process may have multiple benefits from the perspective of the participant. It leaves more time to interact with the participant, making it more comforting and engaging. T-REX may be particularly beneficial for participants who are anxious or nervous about participating. Furthermore, a streamlined process conveys more professionalism and may improve participation satisfaction, ultimately increasing the willingness to participate in future research. Moreover, if the start and recording of experiments are simplified enough, participants may be able to run experiments themselves. The introduction of engaging and fun experiments that enable participants to run them as they like provides the participants with an opportunity to alleviate boredom and do something meaningful by contributing to scientific research. Together, both the researcher (more data) and the participant (more engagement) are benefitted. While T-REX has been developed with independent recording in mind, it is currently not being tested for that purpose.

In comparison with other available software platforms, T-REX is the only solution specifically focused on recording experiments, allowing it to remain lightweight. Platforms like BCI2000 [7], OpenViBE [8], and MEDUSA [11] offer comprehensive functionalities spanning the 3 stages of a BCI system: signal acquisition, signal processing, and feedback presentation. However, they require complete software installation even if only the recording module is needed. T-REX enhances the researcher experience by offering flexibility in the choice of programming language and technology for creating the experiments, unlike BCI2000 and OpenViBE, which mandate the use of C++; MEDUSA, which requires the use of Python; and NFBlab [10], which requires the use of its graphical UI. Regarding compatibility, T-REX holds a distinct advantage, supporting all major OSs, including Windows, Linux, and macOS. This is in contrast with BCI2000’s limited functionality outside Windows and MEDUSA’s exclusive Windows availability, as well as the system presented by Ashmaig et al [12], which is Linux-bound. Each of these platforms has its strengths and excels in its intended function. T-REX provides a tailored solution for a specific part of neuroscientific research that allows it to remain simple and lightweight.

T-REX aims for simplicity, and setting up experiments in T-REX requires basic knowledge of command line interface usage. Moreover, experiments and devices must use LSL to make data available. Although LSL is available for all mainstream OSs and programming languages, experiments already used by researchers may require adjustments to the experiment code structure for inclusion in T-REX. Therefore, technical knowledge and usage of LSL may limit the applicability for some labs. Furthermore, T-REX is available for all mainstream OSs but may not apply to all different versions. Specifically, the command line interface version of LabRecorder, including the script that records and stores the multiple data streams, had to be built for different chipsets (M1 and M2) for macOS. These are currently included, but other architectures likely require a different build of LabRecorder. As T-REX matures, we expect more versions to become applicable.

T-REX is in ongoing development, and we have identified several potential future updates targeting an improved user experience. Device streams currently need to be started manually, and this may be performed automatically at the start of an experiment. This is also a requirement to enable participants to start recordings themselves, which is a main future improvement. Aside from ensuring that there are no manual actions except starting the experiments, allowing T-REX for independent use may require improved internal logging and error handling. Combined, these updates would reduce even more actions for both the researcher and participant, and increase the robustness of T-REX.

In conclusion, T-REX offers a flexible solution to record neuroscientific experiments. It streamlines setup and recording, and reduces error rates that increase the time spent on recordings. We envision T-REX to help standardize and simplify recording experiments and eventually allow recordings by participants independently. This may improve the overall satisfaction of participation and increase the amount of data collected. The open-source nature of T-REX is in the spirit of open science and increases its value through an increase in community knowledge.

## Appendix

Multimedia Appendix 1 The directory tree illustrates the content of the ./output/ folder when saving the experimental data gathered with one experiment. The output.xdf file is created upon experiment completion. It contains the recorded data from the preconfigured Lab Streaming Layer streams. The feedback.txt file contains the feedback the participant inputted on the Experiment Feedback window, and it is saved in the same folder as the most recent .xdf file.

Multimedia Appendix 2 The system-wide configuration file that must be placed inside the root folder of the project, which allows the researcher to configure the execution of T-REX.

Multimedia Appendix 3 Example of the main configuration file. Note that all paths are relative to the main parameter.

Multimedia Appendix 4 The different options for the experiment configuration file. Each experiment must include this file. The parameter command might need to be modified when porting the platform to a different operating system (from Windows to Linux or macOS, for example). It is up to the researcher to perform the redefinition.

Multimedia Appendix 5 Experiment configuration file used for the grasping experiment. This experiment presents simple instructions to the participant indicating continuous opening and closing of either the left or right hand. The visual interface was built using the Python Tkinter library.

Multimedia Appendix 6 Experiment configuration file used for the grasping web experiment. This experiment presents simple instructions to the participant indicating continuous opening and closing of either the left or right hand. The visual interface was built using HTML, CSS (Bootstrap5 for responsiveness and other visual aspects), and JavaScript for behavior.

Multimedia Appendix 7 Experiment configuration file used for the 3D hand-tracking experiment. The goal of the experiment is to hold the cursor on the target. The cursor can be moved in 3 dimensions, where the third dimension controls the size of the circle. In this case, the hand tracking is done by the LeapMotion controller.

Multimedia Appendix 8 The directory tree illustrates a system with 3 different folders, each for a different experiment (∼/EXPERIMENT_1/, ∼/EXPERIMENT_2/, and ∼/EXPERIMENT_3/). Each experiment contains its own configuration file (config.yaml). The researcher can add any additional files to each folder.

Multimedia Appendix 9 Template that can be used for creating an experiment configuration file.

## Abbreviations

EEG: electroencephalography
LSL: Lab Streaming Layer
OS: operating system
T-REX: Standalone Recorder of Experiments
UI: user interface
YAML: Yet Another Markup Language

## Data Availability

The source code, installation guide, and example experiments can be found on GitHub [48]. T-REX is available under the permissive MIT License. As T-REX will be in ongoing development, we kindly invite researchers to provide feedback or contribute to this open-source project.
