# Supplementary figures and images for: The Easy and Versatile Neural Recording Platform (T-REX): Design and Development Study
Source: JMIR Neurotechnol. 2023 Oct 24;2:e47881. doi: 10.2196/47881 (PMC12671289; doi:10.2196/47881)

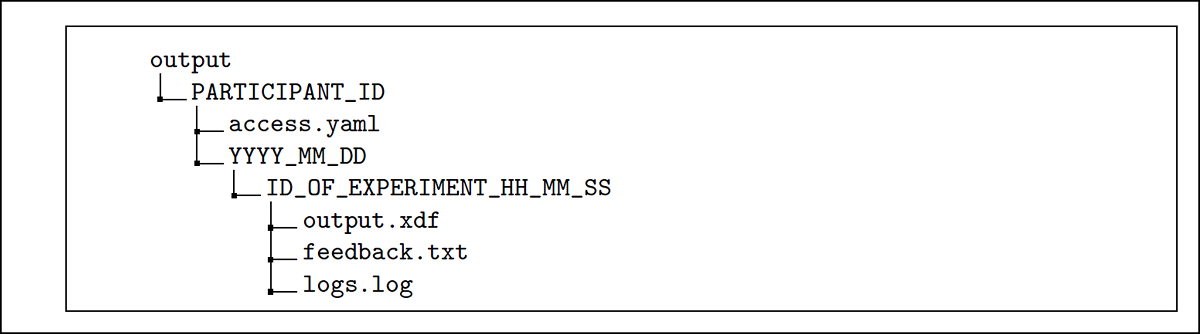

Supplement: Multimedia Appendix 1 [file neuro_v2i1e47881_app1.png]

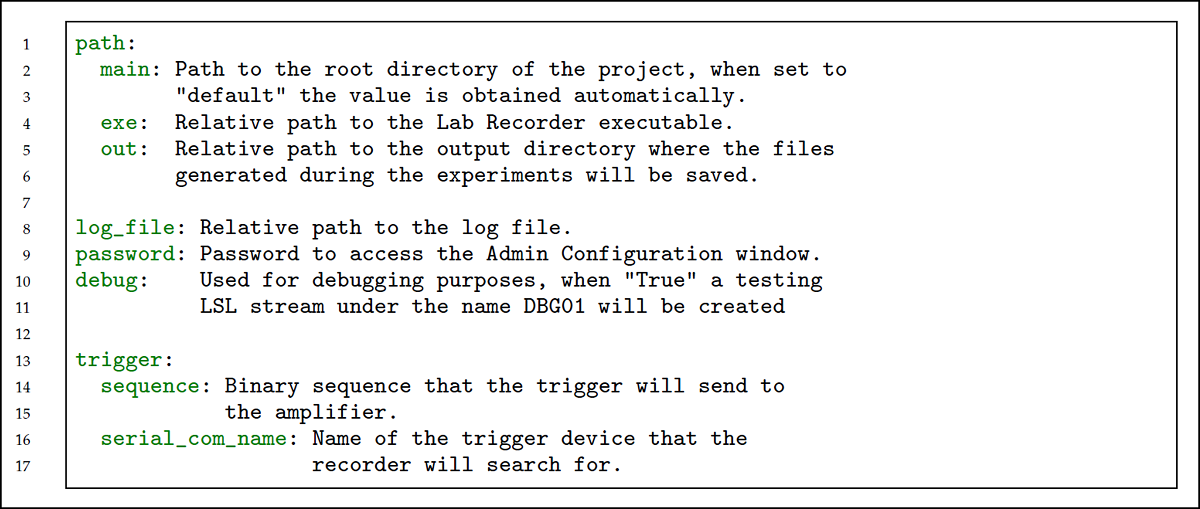

Supplement: Multimedia Appendix 2 [file neuro_v2i1e47881_app2.png]

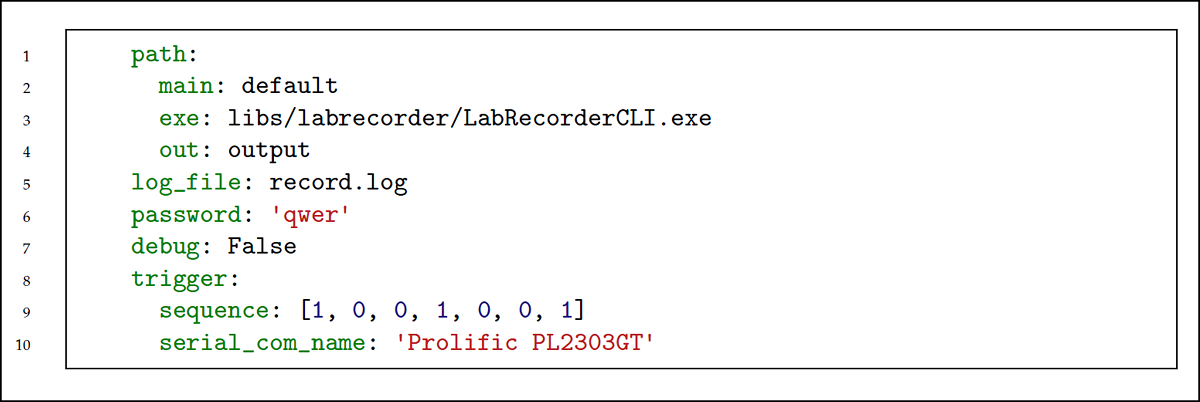

Supplement: Multimedia Appendix 3 [file neuro_v2i1e47881_app3.png]

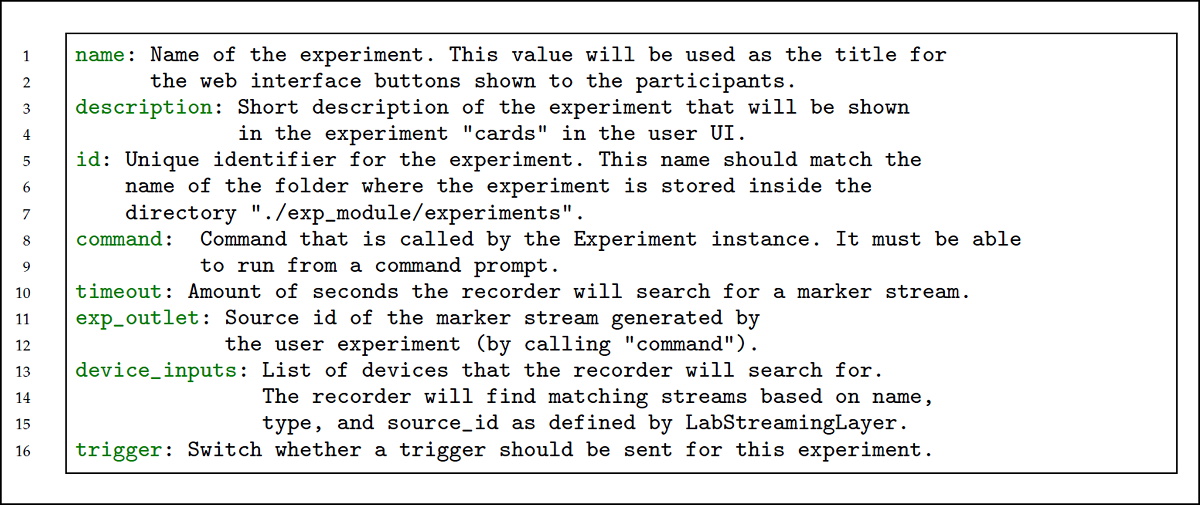

Supplement: Multimedia Appendix 4 [file neuro_v2i1e47881_app4.png]

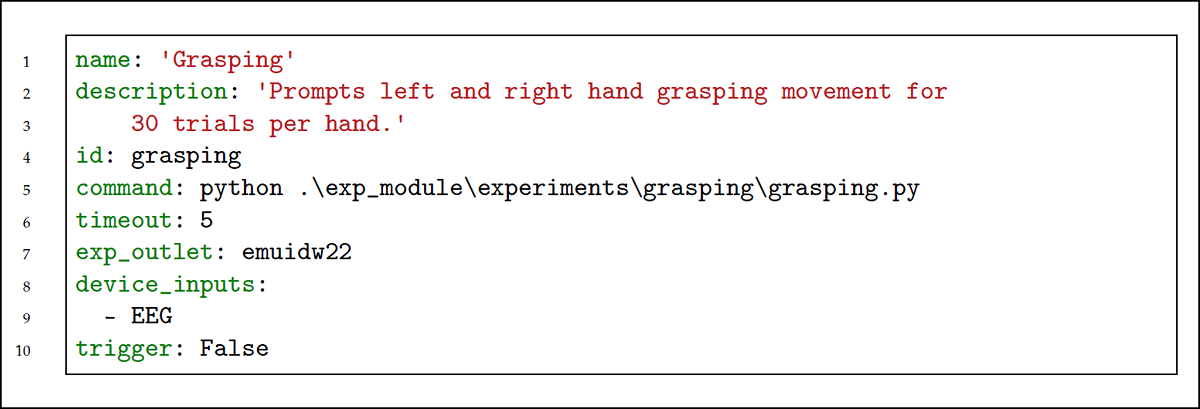

Supplement: Multimedia Appendix 5 [file neuro_v2i1e47881_app5.png]

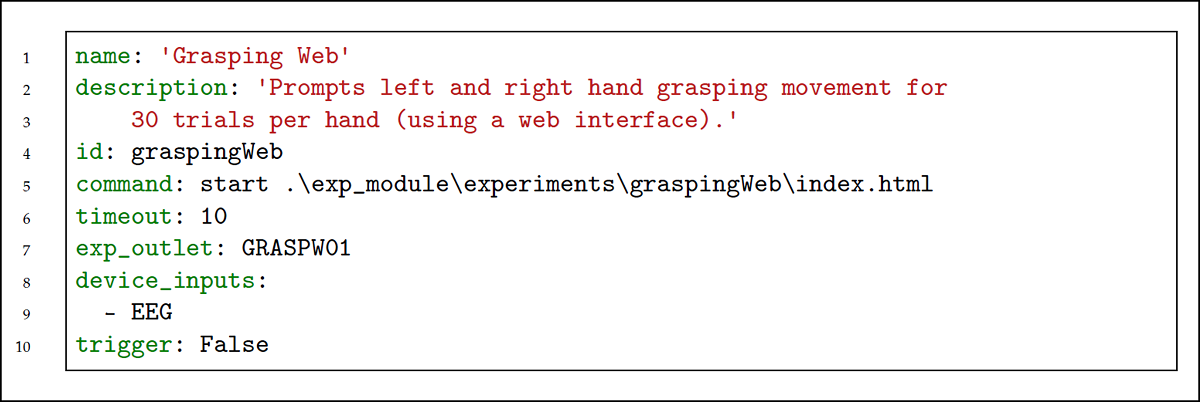

Supplement: Multimedia Appendix 6 [file neuro_v2i1e47881_app6.png]

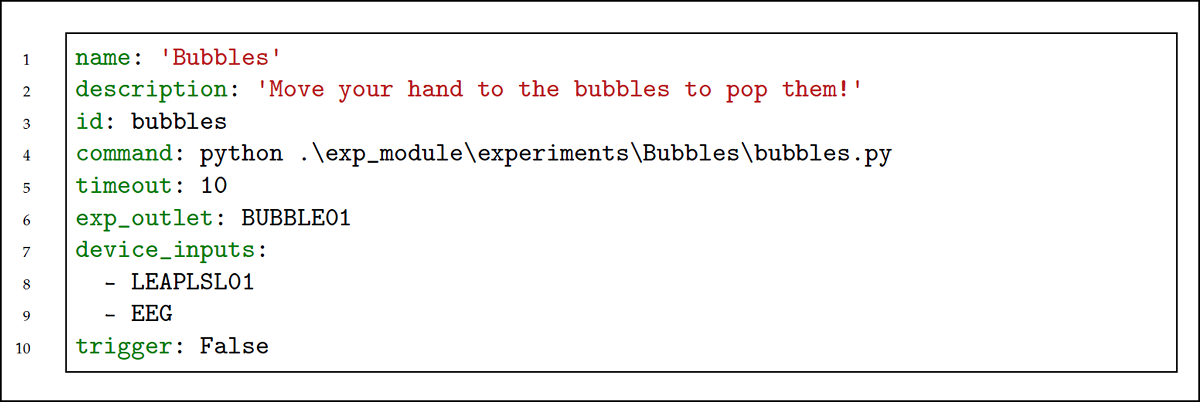

Supplement: Multimedia Appendix 7 [file neuro_v2i1e47881_app7.png]

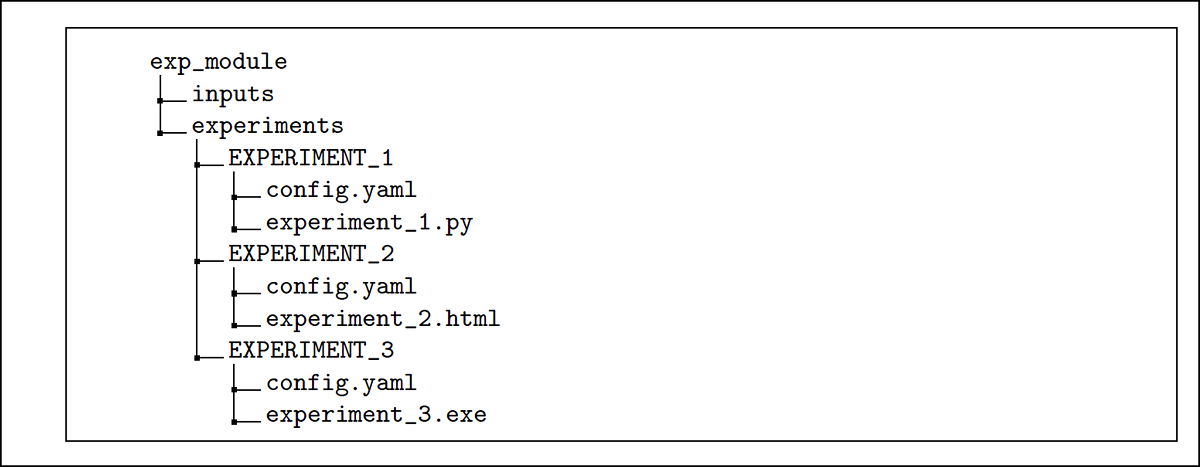

Supplement: Multimedia Appendix 8 [file neuro_v2i1e47881_app8.png]

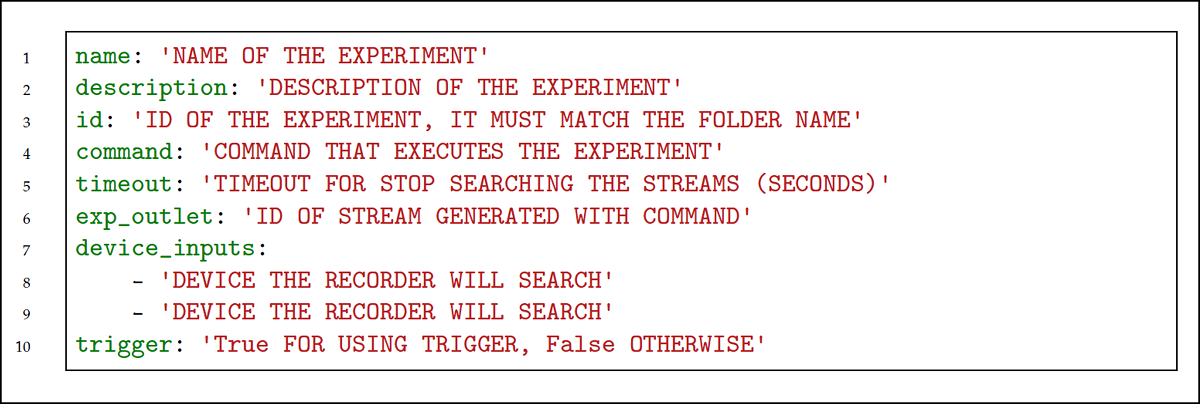

Supplement: Multimedia Appendix 9 [file neuro_v2i1e47881_app9.png]
